# Effects of stimulus-driven synchronization on sensory perception

**DOI:** 10.1186/1744-9081-3-61

**Published:** 2007-12-04

**Authors:** Mark Tommerdahl, Vinay Tannan, Matt Zachek, Jameson K Holden, Oleg V Favorov

**Affiliations:** 1 Department of Biomedical Engineering, University of North Carolina at Chapel Hill, Chapel Hill, North Carolina, USA

## Abstract

**Background:**

A subject's ability to differentiate the loci of two points on the skin depends on the stimulus-evoked pericolumnar lateral inhibitory interactions which increase the spatial contrast between regions of SI cortex that are activated by stimulus-evoked afferent drive. Nevertheless, there is very little known about the impact that neuronal interactions – such as those evoked by mechanical skin stimuli that project to and coordinate synchronized activity in adjacent and/or near-adjacent cortical columns – could have on sensory information processing.

**Methods:**

The temporal order judgment (TOJ) and temporal discriminative threshold (TDT) of 20 healthy adult subjects were assessed both in the absence and presence of concurrent conditions of tactile stimulation. These measures were obtained across a number of paired sites – two unilateral and one bilateral – and several conditions of adapting stimuli were delivered both prior to and concurrently with the TOJ and TDT tasks. The pairs of conditioning stimuli were synchronized and periodic, synchronized and non-periodic, or asynchronous and non-periodic.

**Results:**

In the absence of any additional stimuli, TOJ and TDT results obtained from the study were comparable across a number of pairs of stimulus sites – unilateral as well as bilateral. In the presence of a 25 Hz conditioning sinusoidal stimulus which was delivered both before, concurrently and after the TOJ task, there was a significant change in the TOJ measured when the two stimuli were located unilaterally on digits 2 and 3. However, in the presence of the same 25 Hz conditioning stimulus, the TOJ obtained when the two stimuli were delivered bilaterally was not impacted. TDT measures were not impacted to the same degree by the concurrent stimuli that were delivered to the unilateral or bilateral stimulus sites. This led to the speculation that the impact that the conditioning stimuli – which were sinusoidal, periodic and synchronous – had on TOJ measures was due to the synchronization of adjacent cortical ensembles in somatosensory cortex, and that the synchronization of these cortical ensembles could have been responsible for the degradation in temporal order judgment. In order to more directly test this hypothesis, the synchronized 25 Hz conditioning stimuli that were delivered during the initial TOJ test were replaced with *asynchronous *non-periodic 25 Hz conditioning stimuli, and these asynchronous conditioning stimuli did not impact the TOJ measures.

**Conclusion:**

The results give support to the theory that synchronization of cortical ensembles in SI could significantly impact the topography of temporal perception, and these findings are speculated to be linked mechanistically to previously reported co-activation plasticity studies. Additionally, the impact that such synchronizing conditioning stimuli have on TOJ – which can be measured relatively quickly – could provide an effective means to assess the functional connectivity of neurologically compromised subject populations.

## Background

The perceptual and neuronal responses evoked by single skin site vibratory stimuli have been extensively investigated and reported. As a result, the published literature includes detailed information about how skin mechanoreceptors and individual neurons at all stages of the somatosensory projection pathway from periphery to primary somatosensory cerebral cortex (SI) respond to and represent/encode vibrotactile stimuli (for review see [1]). For example, observations obtained in pioneering neurophysiological recording studies led Mountcastle and colleagues to advance the proposal that a subject's ability to localize a mechanical stimulus on the skin depends on stimulus-evoked dynamic (time-dependent) pericolumnar lateral inhibitory interactions which increase the spatial contrast between regions of SI cortex activated differentially by stimulus-evoked afferent drive [2-4]. Nevertheless, very little information is available about the impact that neuronal interactions evoked by such vibrotactile stimuli at multiple skin sites which project to adjacent and near-adjacent cortical columns could have on sensory information processing.

ATemporal order judgment (TOJ) is a measure obtained from determining the minimal inter-stimulus interval necessary for a subject to detect the temporal order of two sequentially delivered peripheral stimuli. TOJ thresholds have been found to be comparable across all sensory modalities [5], and while several areas of cortex have been implicated as having an important role in timing perception, there is no evidence that somatosensory cortex is directly involved in this temporal process. Timing perception has been shown to be sensitive to lesions to the supplementary motor area (SMA), posterior parietal cortex, and basal ganglia [6,7]. Additionally, these cortical areas have been implicated from significantly above-average TOJ thresholds in subjects with dyslexia [8], dystonia [9-11], and Parkinson's Disease [12].

We sought to take advantage of the fact that SI cortex is most likely not directly involved in the determination of TOJ threshold in order to study the impact of synchronizing sinusoidal stimuli on a subject's ability to spatially distinguish a stimulus. If specific stimulus conditions impact a subject's ability to distinguish between the loci of two temporally separated stimuli, it should also impact a subject's TOJ, but not the subject's temporal discrimination threshold (TDT). In other words, although a subject may detect the presence of two stimuli, he/she may not be able to differentiate the location of those two stimuli under certain conditions of stimulation. Our hypothesis for this study was that if two stimulus sites project to adjacent cortical regions that are functionally linked or bound by a pair of synchronizing stimuli, then the impact that this linkage has on the ability to spatially distinguish two stimuli would impact a subject's TOJ. We tested this hypothesis by comparing TOJ measures obtained at both unilateral and bilateral stimulus sites in the presence and absence of synchronized vibrotactile conditioning stimuli. The results obtained in this study strongly suggest that stimulus-driven synchronization of adjacent cortical ensembles has an impact on a subject's ability to perceptually discriminate between two topographically adjacent skin sites.

## Methods

Twenty subjects (22–32 years in age) were studied who were naïve both to the study design and issue under investigation. All procedures were reviewed and approved in advance by an institutional review board.

A two-alternative forced-choice (2AFC) tracking protocol was used to evaluate the temporal order judgment (TOJ) and temporal discriminative threshold (TDT) capacity of each of 20 right hand dominant subjects. The subject's right arm was rested comfortably on a table surface, and the hand was placed under a portable vibrotactile dual-site stimulator (CM-1; for full description, see [13]). The two probe tips (5 mm diameter each) were positioned at one of three sets of stimulus sites: (1) 30 mm apart along a transversally oriented linear axis along the hand dorsum, (2) on the glabrous pads of digits 2 and 3 of the same hand and (3) on the glabrous pads of digit 2 of both hands. Thus, conditions (1) and (2) were the unilateral conditions and (3) was the bilateral condition. Previous studies have shown that the distance at which the two stimuli were positioned apart on the hand dorsum (30 mm) is well outside a subject's two point discriminative capacity [13-17]. The selection of the two unilateral paired sites allows for not only the comparison of performance at proximal vs. distal skin sites, but allows for the comparison of performance between regions of the skin that are known to have relatively high tactile acuity (digit tips) and regions of the skin that are not (hand dorsum).

**Figure 1:**
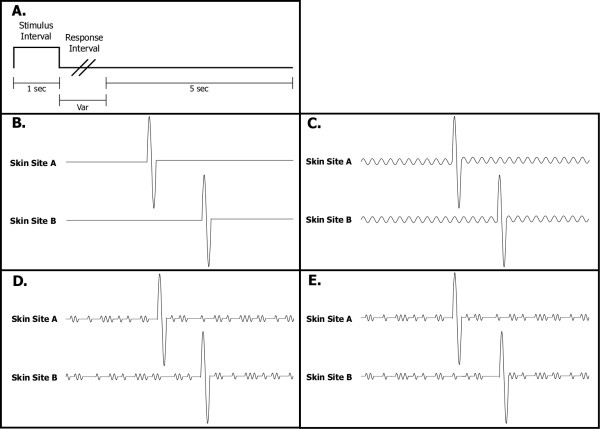
Protocol details. **Panel A: **Two sequential vibrotactile pulses were delivered during the Stimulus Interval, one to each of either skin site A or B. Subject was queried as to which skin site received the first pulse during the Response Interval, and this was followed by a 5 sec delay before the onset of the subsequent trial. **Panel B: **Pulse delivery sequence for the TOJ and TDT tasks during each 1 sec Stimulus Interval. Order of delivery (skin site A or B) was randomized on a trial-by-trial basis, and inter-pulse interval was decreased or increased, depending on subject response. **Panel C: **Exemplary 25 Hz conditioning stimulus delivered concurrently with TOJ/TDT task. **Panel D: **Two non-periodic asynchronous stimuli were delivered concurrently with the TOJ and TDT tests. **Panel E: **Two non-periodic but *synchronous *stimuli were delivered concurrently with the TOJ and TDT tests.

At the start of each run, the two probe tips were driven towards the skin sites until each tip registered a force of 0.1 g, as determined by a closed-loop algorithm in the CM-1 stimulator feedback system. The tips were then further indented into the skin by 500 μm to insure good contact with the skin. The tracking protocol used to obtain individual TOJ and TDT data consisted of 2 separate runs. In the first run (20 trials), two single-cycle vibrotactile test stimuli ("pulses"; 1 mm peak-to-peak amplitude, 25 Hz) delivered to the skin were initially temporally separated by an inter-stimulus interval (ISI) of 150 msec. The locus that received the first of the two pulses was randomly selected on a trial-by-trial basis. The time allocated for stimulus duration was 1 sec (the two 40 ms pulses, separated by the variable ISI, were delivered at the center of this interval), followed by subject response (subject was queried to select the skin site that received the first stimulus) and a 5 sec delay before onset of the next trial (see Panel A of Figure 1). The ISI between the two pulses was modified based on subject response with a 1 up/1 down forced-choice protocol for the first 10 trials and responses for the remaining trials of the run were tracked with a 2 up/1 down forced-choice tracking protocol in which two correct subject responses resulted in a decrement in the ISI. Using a 1 up/1 down tracking protocol for the first 10 trials is an efficient way to quickly move the tracking task into a subject's discriminative capacity range without significantly impacting the results [13]. In the second run (also 20 trials), a 2 up/1 down forced choice tracking protocol was used for TDT assessment. During the stimulus interval, the two pulses were delivered either at the same time or separated temporally by the ISI. Subject response was not dependent on the order of which two stimuli were delivered, but rather on whether the pulses were felt to be simultaneous or not.

Five different conditions of TOJ and TDT assessment were performed. In the first condition, there was no concurrent stimulation (control; see Panel B of Figure 1). In the second and third conditions, a 25 Hz or a 200 Hz concurrent stimulus was delivered, respectively (Panel C of Figure 1). In the fourth condition, two aperiodic asynchronous stimuli were delivered concurrently with the TOJ and TDT tests, and this conditioning stimulus was based on the stimulus used by Romo and colleagues and recently described in Luna et al [18]. In brief, these aperiodic stimuli are composed of fixed length pulses, with one pulse randomly occurring within computationally derived fixed intervals (e.g., for a 25 Hz aperiodic stimulus, one 20 ms pulse is evoked at a random time within each sequential 1/25 sec interval of the stimulus duration). In the fifth condition, two non-periodic but *synchronous *stimuli were delivered concurrently with the TOJ and TDT tests. In all cases of concurrent stimulus delivery, the concurrent stimulus was delivered for a minimum of 400 msec before the first of the two pulses was delivered and lasted for the entire duration of the allotted interval (1 sec) with the exception of the two 40 ms intervals during which the 1 mm pulses were being delivered (compare the non-periodic asynchronous vs. synchronous conditions in Panels D and E of Figure 1).

## Results

**Figure 2:**
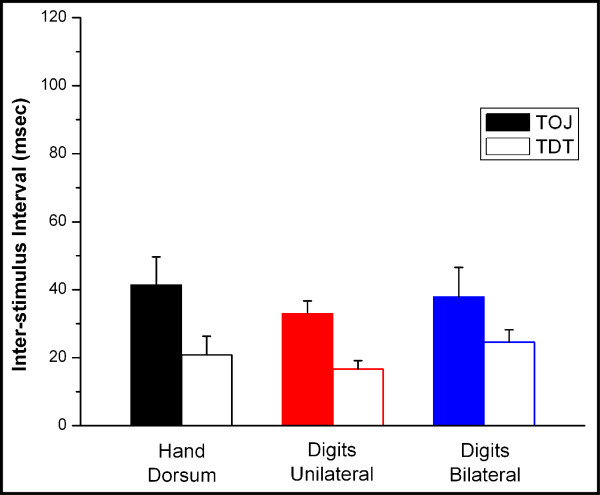
TOJ and TDT measures obtained from 3 different paired skin sites. The TOJ (solid bars) is not significantly different (ANOVA; p > 0.01) across the different sites of stimulation. The TDT (open bars) is not significantly different than TOJ at any of the sites of stimulation (p > 0.01).

A two-alternative forced-choice (2AFC) tracking protocol was used to assess subjects' discriminative capacities to determine the temporal order of two sequentially delivered tactile stimuli (temporal order judgment; TOJ) and to temporally resolve two sequential stimuli, regardless of order (temporal discrimination threshold; TDT). Figure 2 summarizes the TOJ and TDT measures obtained at the selected paired skin sites. The TOJ (solid bars in Figure 2) is not significantly different across the different sites of stimulation (one-way repeated measures ANOVA; p > 0.30 for all TOJ comparisons). Note that TDT (open bars in Figure 2) is significantly lower than TOJ at all respective stimulus sites (p < 0.01 in all cases), but TDT is not significantly different across the different stimulus sites (p > 0.10). Also note that these TOJ and TDT measures fit well within the range recorded by other researchers who obtained the same measures with tactile stimulation [5,9-11,19,20].

**Figure 3:**
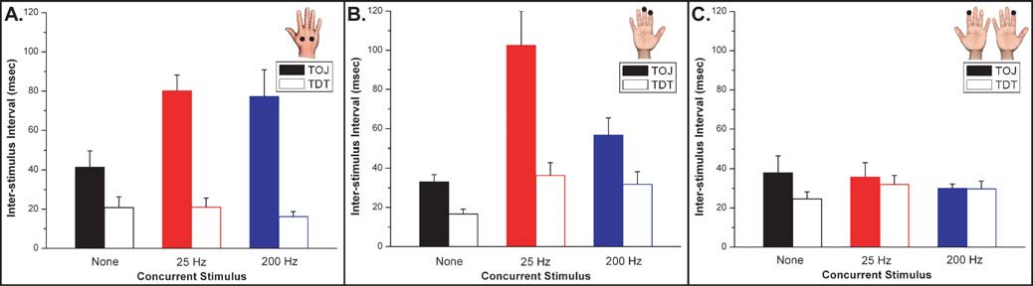
Performance metrics obtained at each of the pairs of stimulus sites under 3 different conditions of concurrent stimulation (0, 25 Hz, and 200 Hz). **Panel A: **Hand dorsum. Note that TOJ thresholds are significantly elevated with the presence of both the 25 Hz and 200 Hz conditioning stimuli (p < 0.01). **Panel B: **Unilateral digit tips 2 & 3. The 200 Hz conditioning stimulus, although it has an impact on the TOJ measure (p < 0.01) does not have as pronounced an effect on the digit tips as on the hand dorsum. **Panel C: **Bilateral digit tips 2. Conditioning stimuli had no significant effect when applied on bilateral digit tips (p > 0.21).

In order to assess whether or not conditioning stimulation would have an impact on TOJ and TDT, conditioning stimuli were delivered before (a minimum of 400 msec) and concurrently with the TOJ and TDT tasks (see Methods). Figure 3 summarizes the TOJ and TDT performance metrics obtained at each of the pairs of stimulus sites for two types of conditioning stimulation (25 Hz and 200 Hz). In each panel, results are plotted for TOJ and TDT measures obtained with 0, 25 Hz and 200 Hz concurrent stimuli. In the unilateral conditions (Figure 3, Panels A & B), in which both stimuli are on the same hand, TOJ thresholds were significantly elevated with the presence of a 25 Hz conditioning stimulus (p < 0.01). The 200 Hz conditioning stimulus – although it clearly has an impact on the TOJ measure in both unilateral conditions (p < 0.01) – does not have as pronounced an effect on the digit tips as on the hand dorsum. Specifically, the TOJ measure with a 200 Hz conditioning stimulus is significantly lower than that with a 25 Hz conditioning stimulus on the digit tips (see Panel B of Figure 3; p < 0.01), but not on the hand dorsum (Panel A; p > 0.44). The different outcomes produced by the 25 Hz vs. 200 Hz conditioning stimuli on the digit tips vs. the hand dorsum could be explained by the difference in PC receptive field sizes at the two locations (i.e., small at the distal tips and relatively large more proximally), and it has been demonstrated in animal imaging studies that such differences in the evoked response to high vs. low frequency stimulation at proximal vs. distal sites could lead to significant alterations in sensory percept. In particular, high frequency (200 Hz) stimulation plays a much more prominent role in SI cortical information processing in more proximal skin regions in a manner consistent with a loss in spatial localization capability (for discussion, see [21]). Regardless of the differences in the responses evoked at the two pairs of unilateral sites, TOJ measures obtained from both of these paired sites were more significantly impacted by the addition of concurrent vibrotactile stimuli than TDT measures obtained at the same sites.

However, very different results were obtained when the two stimuli were located on mirror-opposite digit tips (D2 of the left and right hand). The concurrent vibrotactile stimuli had little or no effect on the TOJ measures obtained in the bilateral stimulus condition (Figure 3, Panel C). In both the 25 Hz and 200 Hz condition, there was no significant difference on a subject's ability to discriminate between the two sites (p > 0.21). The difference in the results obtained between the unilateral and bilateral stimulus conditions clearly shows that there is a significant impact on TOJ when concurrent vibrotactile stimuli are delivered to skin sites that project to cortical regions in the same hemisphere but does not impact the TOJ measure when they are topographically remote.

**Figure 4:**
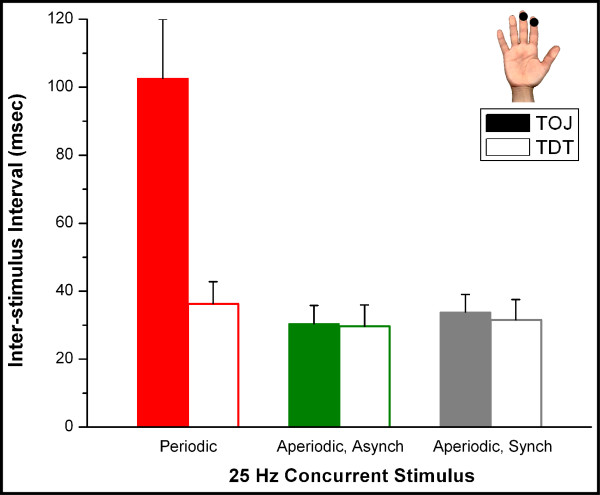
Comparison of periodic vs. nonperiodic stimuli. Replacing synchronized 25 Hz vibrotactile conditioning stimuli (results shown in Figure 3) with asynchronous, nonperiodic stimuli resulted in no significant change from the control condition.

The above described findings led us to consider the hypothesis that synchronization of adjacent or near-adjacent cortical ensembles to which the digit tips project are responsible for the degradation in the TOJ measures shown in Panels A & B of Figure 3. In order to test this idea, another experimental protocol was performed on the same set of subjects in which the synchronized 25 Hz vibrotactile conditioning stimuli were replaced with 25 Hz asynchronous (or synchronous) and non-periodic stimuli (using the stimuli described by Luna et al [18]). Because of the prominent difference of the results obtained on the unilateral digit tips in the presence and absence of conditioning stimulation, we sought to directly compare the effects of periodic vs. aperiodic conditioning stimuli at that pair of stimulus sites (Figure 4). Although TOJ thresholds for the unilateral digits were significantly elevated with the presence of a 25 Hz periodic conditioning stimuli, there was no elevation in the TOJ when non-periodic conditioning stimuli were delivered (either synchronously or asynchronously) concurrent with the TOJ task (p > 0.72).

## Discussion

In this study, we described the impact of conditioning or concurrent vibrotactile stimulation on the ability of a subject to discriminate the temporal order of two stimuli delivered at two different loci on the skin. Our results demonstrate that subjects were able to discriminate the locus of the stimuli when the temporal order differed between 30 and 40 ms in the absence of any conditioning stimulation. When 25 Hz conditioning stimuli were delivered concurrently at both pairs of unilateral test sites, subjects demonstrated a decreased ability to discriminate order of the two sequentially delivered stimuli. All subjects tested demonstrated a decreased ability in TOJ when 25 Hz conditioning stimuli were delivered to both unilateral pairs of skin sites concurrent to the presentation of the sequential pair of test stimuli. Additionally, all subjects tested did *not *show any significant change in TOJ with the same 25 Hz conditioning stimulus in the bilateral condition. While we interpreted the above-described findings to be supportive of the hypothesis that synchronization could be the cause of the degradation in TOJ that was observed in the unilateral 25 Hz condition, we performed a more direct test in which we delivered an asynchronous 25 Hz stimulus to both sites and found that there was no impact – in any subject – on the TOJ with this nonperiodic stimulus condition. We view these findings to be relatively novel in that there have been few, if any, studies on the effects of adaptation or conditioning stimuli on the ability of subjects to assess temporal order, and fewer still on quantitative measures on the perceptual impact of synchronizing stimuli.

While the most significant finding of this study is that synchronous delivery of periodic vibrotactile stimuli to two unilateral regions of the skin that project to adjacent and/or near-adjacent cortical ensembles in SI leads to a significant degradation of a subject's TOJ, it is interesting to note the differences obtained in the TDT and TOJ measures obtained at the digit tips vs. the hand dorsum. Upon examination of the data obtained from the hand dorsum (Figure 3A), it is clear that the TDT measure is totally unaffected by the concurrent conditioning stimuli. Inspection of Figure 3B, however, reveals that there is a clear impact of the same conditions when the test is performed on unilateral D2 and D3 (a 20 ms difference). On the other hand, although TOJ is degraded significantly at both pairs of these sites by the conditioning stimuli, the difference between the degradation (100 ms vs. 80 ms) is approximately equal to the difference in the impact that was on the TDT (40 ms vs. 20 ms). Thus, it appears that there are at least two components of degradation in the TOJ – the temporal component that is evident in the change in TDT exclusively on the digit tips and what could be speculated as a spatial component as evidenced by the change in TOJ under different stimulus conditions on both the digit tips and hand dorsum. Alternatively, it could be speculated that the increased degradation observed at the digit tips over the hand dorsum could be a function of use-dependent plasticity. The finger tips are used together routinely on a daily basis and hence, the functional connection between those associated cortical regions would undoubtedly be much stronger than those connections associated with the hand dorsum. Nevertheless, conditioning or adapting stimuli prior to and concurrent with a TOJ task would, in general, be expected to improve a subject's capacity to spatially distinguish between two stimuli on the skin.

The effects of an adapting stimulus on the perception of subsequent stimuli – particularly the reduction in sensation – has been characterized in some detail [22-27]. However, only a relatively small number of studies have assessed the impact that similar prior or concurrent exposure to vibrotactile stimuli has on spatial localization or the spatial acuity necessary to discriminate between two points on the skin, and all of these studies demonstrated that adaptation *improved *spatial acuity [14,15,28,29]. This improvement was originally proposed to be due to the improved spatial clarity between topographically distinct regions of SI cortical activity [3]. Two recent reports have examined the effects of stimulus duration-dependent changes on a subject's ability to spatially localize a stimulus. Tannan et al [16] demonstrated that the performance of neurologically healthy human adults on a spatial localization task undergoes a prominent change with pre-task exposure to an adapting stimulus. In that study, it was determined that adaptation with a 5 sec vibrotactile stimulus resulted in an approximately 2-fold improvement in spatial localization performance over that achieved with a 0.5 sec adapting stimulus. It was proposed that this observed improvement in spatial localization was due to the enhanced spatial funneling of the population-level response of contralateral primary somatosensory cortex (SI) – a robust phenomenon that is at least in part due to GABAergic inhibitory neurotransmission [30] and has been demonstrated using comparable stimulus conditions in neuroimaging studies of anesthetized non-human primates [31-33]. A subsequent report strengthened this argument by demonstrating that neurologically compromised subjects with a known GABAergic deficiency (adults with autism) showed no such improvement at the same spatial localization task with adaptation [17]. Thus, there seems to be sufficient evidence that spatial acuity does improve in a stimulus-dependent and GABA-mediated manner that undoubtedly impacts the spatial contrast of cortical activity evoked by vibrotactile stimuli. Changes in the responsivity of neurons have been proposed to underlie the cortical mechanisms for stimulus feature extraction and may be important in the improvements observed in spatial discrimination such as those described above (for review see [34]). This enhancement of discrimination capacity could be due at least in part to the moment-to-moment changes that occur in the spatio-temporal patterns of response with repetitive vibrotactile stimulation. In this study, however, spatial acuity apparently became worse in the presence of simultaneously delivered synchronized sinusoidal stimuli – but only when they were applied to the unilateral stimulus sites. Application of the same stimuli to bilaterally opposed digit tips did not result in the same decrease in performance, indicating that the degradation in TOJ performance at the unilateral sites was most likely due to a loss in *temporal *contrast between adjacent and/or near adjacent cortical regions in SI. Delivering non-synchronized 25 Hz stimuli to the same sites during the temporal order judgment task – which did not result in a significant difference from baseline – gives further credence to the idea that spatial acuity is dependent on spatial-temporal integration of cortical activity evoked in SI. Harris et al [35] demonstrated that the topography of tactile working memory could be disrupted (in a frequency discrimination task) if a common cortical region were activated by another stimulus. The findings of this study also suggest that activation of this common cortical territory impacts the topography of temporal perception, but in this case, the timing of the stimulus parameters appears to be critical.

There is a rapidly growing appreciation in neurobiological research of the important contributions to sensorimotor function of coordinated across-neuron patterns of spike discharge activity within the neocortical areas activated by sensory stimuli (for comprehensive review see [36]). In particular, stimulus-induced, time-dependent (dynamic) across-neuron synchronization of action potential discharge and the associated oscillatory modulation of spike firing are common and prominent properties of neocortical networks devoted to the processing of sensory information. The tendency of sensory neocortical networks to generate synchronized oscillations in response to stimulation has raised the possibility that synchronization may play a prominent role in some aspects of sensory perception. For example, in the case of SI cortex, this tendency might impact the ability of SI RA-type neurons to encode the frequency of skin flutter stimulation using a periodicity code [1]. Could synchronization also impact the ability of these same neurons to localize stimuli on the skin? It should be emphasized that the stimulus paradigm that was used in this study was derived with the goal of understanding what the impact of stimulus-driven synchronization has on adjacent cortical ensembles and the spatio-temporal integration of information that results from those ensembles being temporally linked or bound by a common synchronizing input. Clearly, the stimulus-driven linkage between topographically adjacent sites can result in spatial identification errors, most likely because these cortically adjacent regions are being driven with a simultaneous and identical sinusoidal pair of tactile stimuli which contribute to a loss in spatio-temporal contrast required for discrimination between the two sites.

The prominent system of long-range horizontal connections linking cortical columns across 4–5 mm of neocortex is a likely conduit for the synchronizing interactions that occur between SI macrocolumns ([37-40]. Since these connections are provided by axon collaterals of excitatory pyramidal cells terminating on both excitatory and inhibitory neurons in the target cortical columns, their physiological effect on the target columns is not straightforward. For example, when these interlinked cortical columns are both effectively driven from their sensory periphery, they have a predominantly inhibitory effect on each other ([41-46] that would predictably have a negative impact on the subject's capacity to spatially distinguish between the two loci. Other researchers have found that long duration (3 hr) stimulation or "co-activation" of digit tips can lead to improvements in spatial discrimination at the stimulated sites but mislocalizations between skin sites that were synchronously co-activated [47-50]. Additionally, Kalisch et al demonstrated that asynchronous co-activation does not result in the increases in tactile acuity or spatial mislocalizations that is experienced with synchronous co-activation [51]. One interpretation of these co-activation findings is that the strength of the connections between adjacent cortical columns is altered in a Hebbian dependent manner which then leads to an improvement in tactile acuity ([48,49,52-55]. Additionally, it appears that the timing of the co-activation is critical for the initiation of these plastic changes [50,56]. In the case of our particular experimental paradigm, adjacent and/or near-adjacent cortical ensembles would appear to become temporally linked when driven simultaneously, and this temporal or functional linkage, in turn, could lead to mislocalizations between the skin sites that project to those cortical ensembles. Thus, one possibility is that the same functional linkage that is exploited for the above-mentioned co-activation plasticity studies (i.e., 3 hrs of synchronizing stimuli which lead to improvements in tactile acuity and degradations in localization performance) actually makes TOJ performance worse on a much shorter time scale than would be anticipated by these experience-dependent plasticity studies. We anticipate that, in the presence of synchronizing 25 Hz stimuli, the cortical regions that normally respond to one of the pulsed TOJ stimuli now respond to either stimuli at either skin site in partial unison, and a longer inter-stimulus interval between these pulses will be required to have both the spatial and temporal contrast necessary to make a perceptual distinction between the stimuli delivered to these two loci. This interesting possibility, as well as the role of specific neurotransmitter systems involved in this phenomenon, is currently being more directly investigated.

## Conclusion

These results suggest that in healthy adult subjects – in which functional connectivity between adjacent and/or near adjacent cortical columns is intact or not impaired – the TOJ measure will be significantly impacted in the presence of a stimulus which simultaneously engages paired cortical ensembles. Additionally, the impact that such synchronizing conditioning stimuli have on TOJ – which can be measured relatively quickly – could provide a means to assess the degree to which some neurologically compromised subject populations are impaired.

## Abbreviations

TOJ = Temporal Order Judgment

TDT = Temporal Discriminative Threshold

Hz = Hertz

SMA = supplementary motor area

2AFC = two-alternative forced-choice

D2 = digit 2

D3 = digit 3

CM = Cortical Metrics

mm = millimeters

g = gram

msec = milliseconds

sec = seconds

hr = hour

ISI = inter-stimulus interval

ANOVA = analysis of variance

GABA = gamma-aminobutyric acid

## Competing interests

The author(s) declare that they have no competing interests.

## Authors' contributions

MT and VT participated in the design and conduct of the experiments and the drafting of the manuscript. MZ participated in the design and the conduct of the experiments. JH participated in the conduct of the experiments. OF participated in the draft of the manuscript. All authors read and approved the final manuscript.
